# DeepLabV3+-Based Semantic Annotation Refinement for SLAM in Indoor Environments

**DOI:** 10.3390/s25113344

**Published:** 2025-05-26

**Authors:** Shuangfeng Wei, Hongrui Tang, Changchang Liu, Tong Yang, Xiaohang Zhou, Sisi Zlatanova, Junlin Fan, Liping Tu, Yaqin Mao

**Affiliations:** 1 School of Geomatics and Urban Spatial Information, Beijing University of Civil Engineering and Architecture, Beijing 102616, China; weishuangfeng@bucea.edu.cn (S.W.); 2108160323006@stu.bucea.edu.cn (H.T.); liuchangchang1023@163.com (C.L.); zxhang2021@163.com (X.Z.); 2 Research Center of Representative Building and Architectural Heritage Database, Ministry of Education, Beijing 102616, China; 3 GRID, School of Built Environment, UNSW Sydney, Sydney, NSW 2052, Australia; s.zlatanova@unsw.edu.au; 4 Jiangxi Nuclear Industry Surveying and Mapping Institute Group Co., Ltd., Nanchang 330000, China; fanjunlin2000@163.com (J.F.); m18970899190@163.com (L.T.); lemonmyq0805@163.com (Y.M.)

**Keywords:** image semantic segmentation, visual SLAM, point cloud semantic annotation, clustering, scene understanding

## Abstract

Visual SLAM systems frequently encounter challenges in accurately reconstructing three-dimensional scenes from monocular imagery in semantically deficient environments, which significantly compromises robotic operational efficiency. While conventional manual annotation approaches can provide supplemental semantic information, they are inherently inefficient, procedurally complex, and labor-intensive. This paper presents an optimized DeepLabV3+-based framework for visual SLAM that integrates image semantic segmentation with automated point cloud semantic annotation. The proposed method utilizes MobileNetV3 as the backbone network for DeepLabV3+ to maintain segmentation accuracy while reducing computational demands. In this paper, we introduce a parameter-adaptive Density-Based Spatial Clustering of Applications with Noise (DBSCAN) clustering algorithm incorporating K-nearest neighbors and accelerated by KD-tree structures, effectively addressing the limitations of manual parameter tuning and erroneous annotations in conventional methods. Furthermore, a novel point cloud processing strategy featuring dynamic radius thresholding is developed to enhance annotation completeness and boundary precision. Experimental results demonstrate that our approach achieves significant improvements in annotation efficiency while preserving high accuracy, thereby providing reliable technical support for enhanced environmental understanding and navigation capabilities in indoor robotic applications.

## 1. Introduction

Autonomous robotic navigation systems have emerged as a critical component in artificial intelligence research, attracting significant attention in recent years. These systems typically rely on visual sensors (e.g., RGB-D cameras) to acquire real-time environmental data and employ Simultaneous Localization and Mapping (SLAM) technology for three-dimensional scene reconstruction [1]. However, conventional SLAM systems produce maps that are purely geometric, devoid of higher-level semantic understanding, which significantly hinders robots’ capacity to execute intelligent interaction tasks, such as object grasping and obstacle avoidance, within intricate indoor environments [2]. Consequently, the construction of high-precision semantic maps has become one of the fundamental challenges in advancing autonomous robotic navigation capabilities.

Considering these limitations, researchers have turned to exploring automated semantic annotation methods for point clouds, aiming to bolster both efficiency and accuracy. Early segmentation models were constrained by fixed-size inputs and limited resolution recovery capabilities. The transition from fully convolutional networks to multi-scale feature fusion marked significant progress. Fully Convolutional Networks (FCN) improved traditional Convolutional Neural Networks (CNN) [3] by replacing fully connected layers with convolutional layers. This allowed for the first time to perform segmentation on images of varying sizes without resizing. Although their restricted receptive fields frequently resulted in ambiguous boundary segmentation, subsequent research introduced encoder–decoder architectures like SegNet. These frameworks utilized pooling indices to regain spatial details, mitigating the limitations of low-resolution images; however, segmentation of small objects continued to pose a challenge [4,5,6]. Zhao et al. introduced the Pyramid Pooling Module (PPM) [7,8], which significantly enhanced segmentation accuracy for objects of diverse sizes by integrating multi-scale contextual information, albeit with an associated increase in computational complexity. To strike a balance between accuracy and computational efficiency, Poudel et al. [9] developed Fast-SCNN. This network features a lightweight dual-branch architecture that achieves competitive segmentation accuracy while facilitating real-time inference. Nonetheless, the network’s optimal balance between boundary detail and computational efficiency remains an area that calls for further refinement. An additional development was PIDNet, introduced by Xu et al. [10]. This network incorporates parallel branches for detail and semantic information, both equipped with attention mechanisms. This design aims to enhance the quality of boundary segmentation while optimizing the speed-accuracy trade-off. Nevertheless, models often lacked suboptimal robustness under extreme lighting variations or severe occlusion situations. In 2023, Zhang et al.’s MP-Former [11] demonstrated accelerated convergence and maintained high segmentation accuracy through an enhanced Transformer architecture. MobileViG [12], proposed by Munir et al., offered computational efficiency advantages but compromised deep semantic feature extraction capabilities. FusionFormer [13], introduced by Cai et al., combines RGB and point cloud features; despite this innovative approach, it is burdened with a high computational complexity. Sang et al. [14] introduces a novel cross-dimensional scene representation module within the reinforcement learning framework. This module effectively extracts features using both 2D and 3D observation encoders, and it incorporates a joint representation network that enhances and aligns these features across dimensions, thus enabling the seamless fusion of 2D and 3D information. Both approaches have not effectively resolved the challenge of semantic consistency loss in the 2D-3D projection process. While these methods may perform well within their specific areas of application, they collectively encounter challenges related to the processing of point cloud data’s high dimensionality. As the literature notes [15], the computational demands for point cloud processing can be three to five times greater than for image processing. This can lead to limitations such as insufficiently large receptive fields, boundary ambiguities, and difficulties in meeting real-time performance requirements. In contrast, DeepLabV3+ [16], with its Atrous Spatial Pyramid Pooling (ASPP) module for multi-scale context fusion, proves more suitable for semantic annotation tasks in complex indoor environments.

Responding to the challenges, this study presents an innovative framework for automatically optimizing semantic annotations on visual SLAM point clouds using DeepLabV3+. The framework is designed around the inclusion of a fine-grained semantic analysis module within the keyframe stages of the SLAM process. This module utilizes a sophisticated deep convolutional neural network to achieve highly accurate classification of scene elements. To enhance this classification, the framework incorporates depth information from RGB-D sensors. This allows it to map two-dimensional semantic data onto a three-dimensional space, resulting in the generation of a point cloud model with semantic annotations. This significantly improves boundary accuracy. The heart of this technological advancement is an adaptive DBSCAN clustering parameter optimization mechanism. This mechanism removes the need for manual parameter tuning, a feature commonly found in traditional methods. It dynamically adjusts eps and MinPts values in real-time, optimizing the clustering process and improving accuracy. The adaptive DBSCAN mechanism and the segmentation module work together in a symbiotic manner. The optimized DBSCAN parameters set the regional growth threshold, while the refined semantic segmentation provides increasingly reliable prior information. This creates a self-improving feedback loop that continually refines the framework’s performance. By integrating this dynamic approach, our framework eliminates the drawbacks of manual parameterization and facilitates a closed-loop improvement process. This iterative enhancement significantly boosts the overall quality of semantic labeling in the framework. Our experimental findings indicate that this optimization strategy not only significantly enhances the accuracy of point cloud segmentation but also greatly diminishes the necessity for manual adjustments.

## 2. Materials and Methods

### 2.1. Overview of Research Methodology

**Figure 1:**
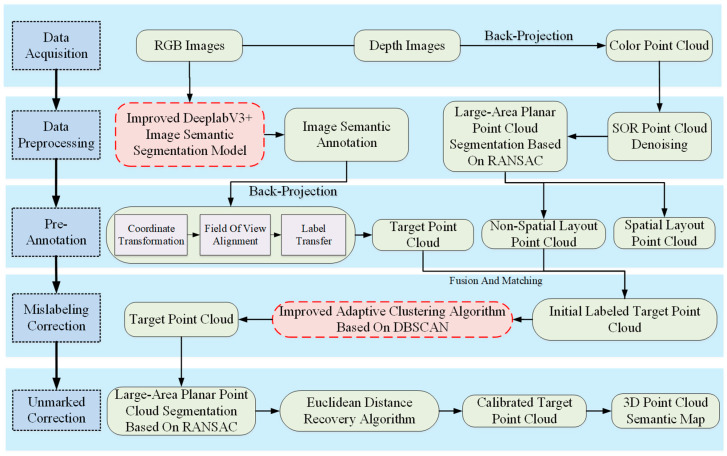
Flowchart of the optimization method for automatic semantic annotation of indoor point clouds proposed in this paper.

This study adopts a systematic processing pipeline, as depicted in Figure 1, which encompasses five integral and interconnected processing phases. The workflow commences with the acquisition of raw data using specialized sensing equipment designed to ensure a comprehensive environmental perception. Following this, the system undergoes data preprocessing, which employs adaptive filtering and geometric correction algorithms to refine the raw data, thereby offering a high-quality input for the subsequent analysis phase. Utilizing the preprocessed data, the system then performs automatic semantic annotation via deep neural networks, which serves to provide an initial identification and classification of scene elements. To rectify inherent errors present in the initial annotations, the system employs density-based spatial clustering algorithms to carry out refinement correction, leading to a significant enhancement in annotation accuracy. Finally, a region-growing-based completion mechanism is applied to identify and supplement missing annotations, ensuring the completeness and reliability of final annotation results.

The proposed methodology follows a systematic processing pipeline consisting of five key phases, as detailed below:

(1) Data Collection and 3D Reconstruction: Utilizing RGB-D sensor-based SLAM technology, we collect scene color and texture information. This technology integrates RGB and depth images to produce 3D colored point clouds, which are then used for scene reconstruction.
(2) Data Processing and Optimization: The data processing phase leverages the DeepLabV3+ architecture to identify and segment objects within RGB images. Concurrently, the Random Sample Consensus (RANSAC) algorithm is employed to detect and segment extensive planes from the point clouds. Furthermore, the Statistical Outlier Removal (SOR) algorithm is utilized for data denoising, resulting in the generation of superior-quality colored point clouds.
(3) Pre-Annotation Processing: Semantic information from 2D images is mapped onto 3D point clouds, and labels are transferred to produce semantically annotated point clouds. Subsequently, planar point clouds identified using the RANSAC algorithm are integrated with spatial point clouds to ensure the completeness of the dataset.
(4) Mislabeling Correction: An improved adaptive DBSCAN clustering algorithm corrects initial annotation errors.
(5) Unlabeled Data Processing: We reapply the RANSAC algorithm for the segmentation of large-scale planes and denoising. This is followed by the regeneration of the target point cloud using Euclidean distance calculations, which retrieves unlabeled points and guarantees the completeness of the semantic annotation.

### 2.2. Visual SLAM System with Semantic Segmentation

**Figure 2:**
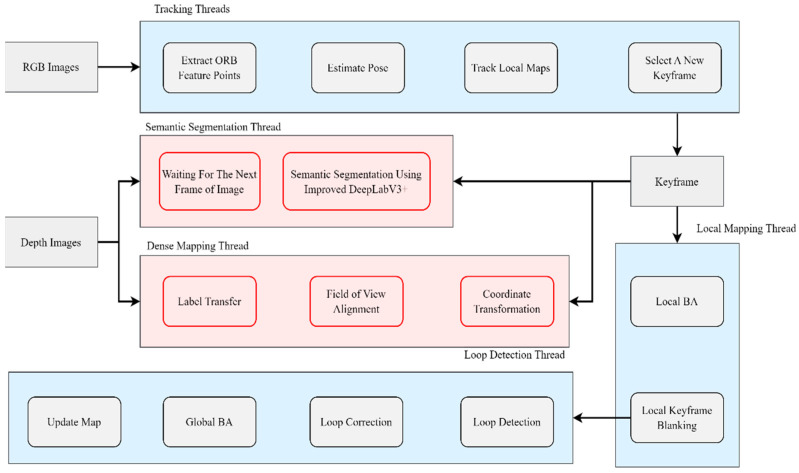
SLAM framework diagram.

In the initial phase of our research, we concentrate on environmental data acquisition and preliminary semantic annotation through the visual SLAM system. To achieve this, we propose a novel visual SLAM framework that combines ORB feature points with a DeepLabV3+ network model, significantly augmenting the semantic segmentation and dense mapping capabilities of the original ORB-SLAM2 architecture [17]. Figure 2 visually depicts the system’s architecture, where RGB and depth image streams are processed concurrently as parallel inputs. The system leverages multi-threaded processing, which ensures both efficiency and robustness in simultaneous mapping and localization tasks.

The system initialization involves a tracking thread that continually estimates camera pose by extracting and matching ORB feature points from incoming RGB images. This first pose estimation is then refined through local map matching to enhance tracking robustness. Concurrently, a separate semantic segmentation thread operates, using the DeepLabV3+ model for pixel-level semantic labeling of keyframe images. This parallel architecture allows for real-time segmentation without compromising system responsiveness. Meanwhile, a dense mapping thread processes synchronized keyframe-depth pairs to build detailed 3D representations of the environment. The architecture includes a closed-loop detection and global optimization module, which triggers upon loop closure detection, performing comprehensive error correction via (1) local bundle adjustment for immediate pose correction, (2) global bundle adjustment to correct accumulated drift, and (3) map updating to preserve topological consistency.

### 2.3. Image Semantic Segmentation Network Based on DeepLabV3+

**Figure 3:**
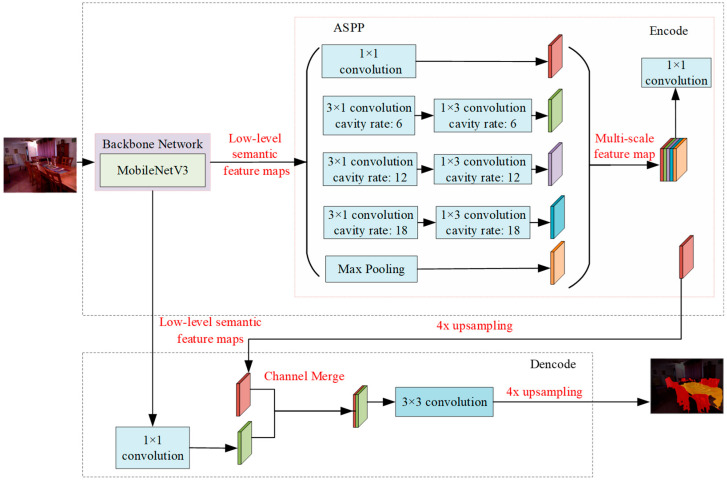
DeepLabV3+ network architecture.

The DeepLabV3+ model, based on an encoder–decoder framework [12,16], is an advanced semantic segmentation tool. The encoder component leverages a feature extraction network in conjunction with an innovative Atrous Spatial Pyramid Pooling (ASPP) module, which is designed to extract spatial, spectral, and textural details at multiple scales. The ASPP module consists of five parallel branches, including a standard 1 × 1 convolution and three dilated convolutions with dilation rates of 6, 12, and 18, enabling multi-scale spatial analysis. Additionally, this global average pooling layer captures crucial contextual information essential for accurate segmentation [18,19,20,21,22]. To address the challenges of model complexity and high memory consumption—particularly for deployment in resource-constrained SLAM systems—we implement DeepLabV3+ with a MobileNetV3 [23,24] backbone for RGB image semantic extraction. This optimized configuration achieves a reduction in network parameters. Figure 3 provides an overview of the DeepLabV3+ network architecture.

### 2.4. Semantic Annotation Optimization Method for Indoor Scene Point Clouds

Deep learning-based semantic segmentation often results in over-segmentation and under-segmentation, which can lead to significant annotation errors when 2D segmentation results are transferred to 3D point clouds. To overcome this critical issue, we have developed an optimized point cloud annotation refinement method. This method systematically processes 3D point clouds through two distinct phases after removing large planar regions (walls, floors, etc.) from indoor scenes. The first phase involves density-based clustering to correct mislabeled object point clouds. The second phase employs Euclidean distance-based completion to fill in unannotated target point clouds. We have rigorously evaluated this optimized post-processing algorithm on the S3DIS indoor point cloud dataset [25]. The results indicate that the method effectively improves segmentation accuracy across various performance metrics, including accuracy, precision, recall, and F1-score.

#### 2.4.1. KD-Tree Accelerated Adaptive DBSCAN for Point Cloud Semantic Correction

DBSCAN is a well-known density-based clustering algorithm. It is recognized for its capacity to detect clusters as density-connected sample clusters, its robustness to noise, and its ability to identify clusters of any shape. The clustering performance of DBSCAN is heavily influenced by two key parameters: the neighborhood radius (eps) and the minimum point threshold (MinPts). Traditional DBSCAN implementations require manual parameter specification, which can be challenging. High MinPts values may lead to the omission of small clusters, while low values introduce noise contamination. Similarly, an oversized eps radius risks noise misclassification, whereas an undersized value induces cluster fragmentation, with computational complexity additionally scaling with dataset size. To address these limitations, we propose an optimized DBSCAN framework that automatically adjusts the parameters eps and MinPts based on the dataset’s inherent statistical properties. This approach obviates the need for manual parameter tuning. Additionally, the framework utilizes KD-tree structures to accelerate near-neighbor searches, thereby improving computational efficiency.

(1) Automatic determination of the eps parameter

Ensuring the rationality of the number of clusters following partitioning requires the identification of an optimal critical threshold. We utilize the K-Average Nearest Neighbor (KANN) method and the mathematical expectation approach to generate a candidate list for eps values [26]. The KANN method works by calculating the distance from each object point in the dataset D to its K-th nearest neighbor and then averaging these distances to determine the KANN distance for the dataset. When K = 1, the KANN distance corresponds to the average nearest-neighbor distance. The KANN distance vector is constructed by calculating the KANN distances for various values of K. The detailed procedure for this method is as follows:

The distance distribution matrix of dataset D is computed as follows:

(1) $$D_{n\times n}=\{dist(i,j)|1\leq i\leq n,1\leq j\leq n\}$$

Let $D_{m\times n}\in R^{n\times n}$ be a real symmetric matrix, where $n=\left|D\right|$ denotes the cardinality of dataset *D*. Each matrix element dist(i,j) represents the distance between the *i*-th and *j*-th objects in *D*, computed using the Euclidean distance metric as defined in Equation (2):

(2) $$dist(i,j)=\sqrt{{\left(x_{i}-x_{j}\right)}^{2}+{\left(y_{i}-y_{j}\right)}^{2}+{\left(z_{i}-z_{j}\right)}^{2}}$$

For the distance distribution matrix $D_{n\times n}$, each row of values is sorted in ascending order. Let $D_{n\times k}$ represent the *k*-th smallest distance value for each of the n object points after sorting. The elements in the first column form a distance vector $D_{0}$ where all values are zero, representing the distance from each object point to itself. The elements in the *k*-th column constitute the nearest neighbor distance vector $D_{k}$ for all object points. The average of elements in vector $D_{k}$ is calculated as shown in Equation (3).

(3) $${\bar{D}}_{k}=\frac{1}{n}\sum_{k=1}^{n}D_{k}$$

The KANN distance $\bar{D_{k}}$ derived from vector $D_{k}$ serves as a candidate value for the eps parameter. By computing the KANN distances for all values of k, we obtain the eps parameter list $Eps_{k}$, as formalized in Equation (4):

(4) $$Eps_{k}=\{{\bar{D}}_{k}|1\leq k\leq n\}$$

**Figure 4:**
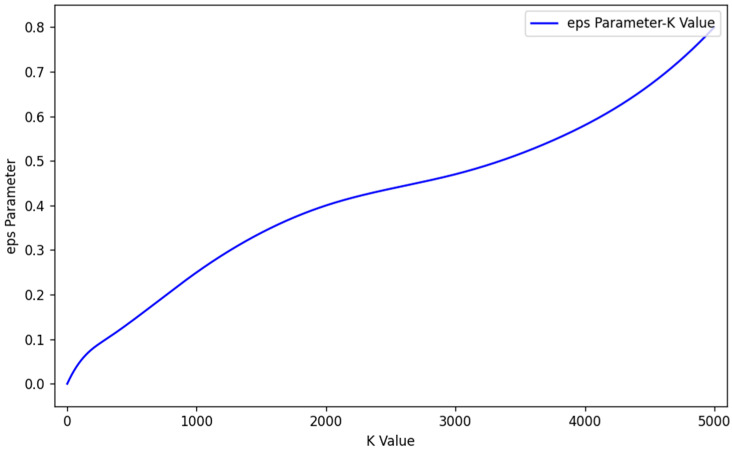
Eps parameter graph.

Figure 4 presents the variation curve of the eps parameter in relation to K values for a test dataset. On the horizontal axis, K values are plotted, while the vertical axis shows the corresponding estimated eps values. Notably, as K increases, the estimated eps values demonstrate a consistent upward trend.

(2) Determination of MinPts Parameter

This study primarily focuses on 3D point cloud data. To achieve more accurate clustering results, the MinPts value is typically set to be greater than or equal to 4. After determining the candidate eps parameter list $Eps_{k}$, we count the number of neighboring points within the eps-radius for each object point in dataset D. The MinPts parameter is then derived by computing the mathematical expectation of the number of eps-neighborhood points across all object points in the dataset. The calculation formula for MinPts is given by Equation (5):

(5) $$MinPts_{k}=\frac{1}{n}\sum_{i=1}^{n}p_{i}$$

**Figure 5:**
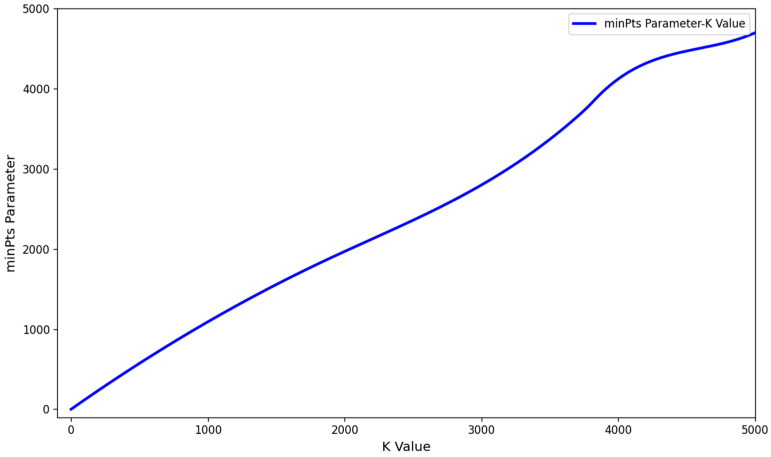
MinPts parameter graphs.

In this formulation, $p_{i}$ denotes the number of object points within the eps-neighborhood of the i-th object point.

(6) $$p_{i}=count\left\{dist(i,j)\leq Eps_{k}|1\leq j\leq n,1\leq k\leq n\right\}$$

where n denotes the total number of object points in dataset D. Following this procedure, we ultimately obtain the $MinPts_{k}$ parameter list corresponding to the $Eps_{k}$ candidate list. As illustrated in Figure 5, which shows the variation curve of MinPts estimates versus their corresponding K values, it can be observed that the MinPts estimates increase steadily with growing K values.

Through the aforementioned methodology, candidate lists for the two critical parameters $Eps_{k}$ and $MinPts_{k}$ are obtained. To identify adaptive optimal parameters suitable for diverse datasets, this study sequentially substitutes these estimated candidate values into the adaptive DBSCAN algorithm. By automatically detecting intervals where the number of clusters remains stable during parameter variation, the optimal parameters are selected as the pair (eps, MinPts) corresponding to the minimum density threshold within the stable interval [27]. This approach achieves complete automation without requiring manual intervention.

#### 2.4.2. Unannotated Point Cloud Correction Based on Euclidean Distance Objective

Unannotated points in point clouds refer to points that should belong to a specific category but were not correctly labeled (i.e., missing annotations), while still maintaining close spatial relationships with the target point cloud. Building upon the method proposed in [28], we implement a recall strategy based on Euclidean distance metrics to achieve point cloud regrowth, thereby accomplishing semantic annotation correction.

For the target point cloud $D=\left\{P_{i}\left|0\leq i\leq numLabel\right.\right\}$, where *numLabel* represents the number of label categories and *ClustetID* is assigned to distinct objects of the same category, the neighborhood thresholds $r_{1}$ and $r_{2}$ are configured to minimize manual intervention while adapting to each category’s geometric characteristics. The threshold $r_{1}$ is directly derived from the optimal eps value obtained through the adaptive parameter estimation method in Section 2.4.1. For $r_{2}$, we first identify the extremum coordinates of each category’s point cloud along the *x*, y, and *z* axes, then compute the maximum Euclidean distance between these extrema using Equation (7). This automated approach ensures $r_{1}$ reflects local density properties while $r_{2}$ captures global category-wise spatial distribution, with both thresholds dynamically adjusted to the dataset’s inherent structure.

(7) $$maxdist=\sqrt{{\left(x_{max^{-}}-x_{min^{-}}\right)}^{2}+{\left(y_{max^{-}}-y_{min^{-}}\right)}^{2}+{\left(z_{max^{-}}-z_{min^{-}}\right)}^{2}}$$

The neighborhood threshold $r_{2}$ is set to half of the maximum distance.

(8) $$r_{2}=\frac{maxdist}{2}$$

## 3. Results and Discussion

**Figure 6:**
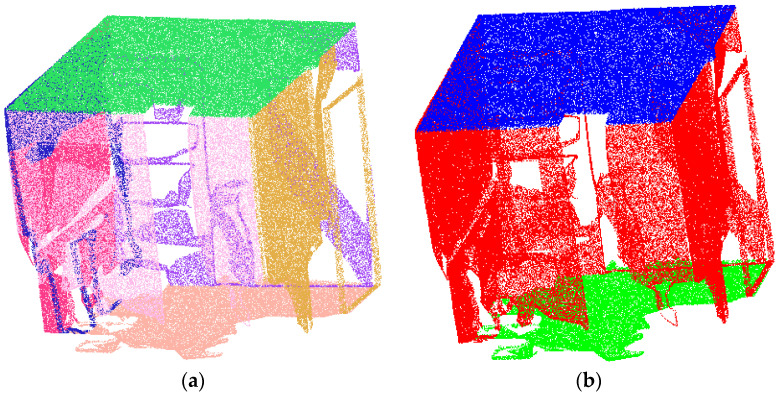
Large area planar point cloud: (**a**) large area planar point cloud segmentation; (**b**) spatial layouts point cloud classification.

This study selected the office_7 environment from Area 2 of the 2D-3D-S dataset as a benchmark scenario to validate our proposed technical approach. After conducting the benchmark tests, we further collected additional real-world scene data to ascertain the method’s generalizability. Preprocessing the office_7 point cloud entails two key steps: First, we utilize RANSAC-based multi-plane segmentation to eliminate large-scale structural elements (such as ceilings, walls, and floors), thereby isolating target objects from the background spatial relationships. Second, we significantly reduce computational load by isolating indoor objects, a process illustrated in Figure 6.

### 3.1. Image Segmentation and Semantic Mapping

**Figure 7:**
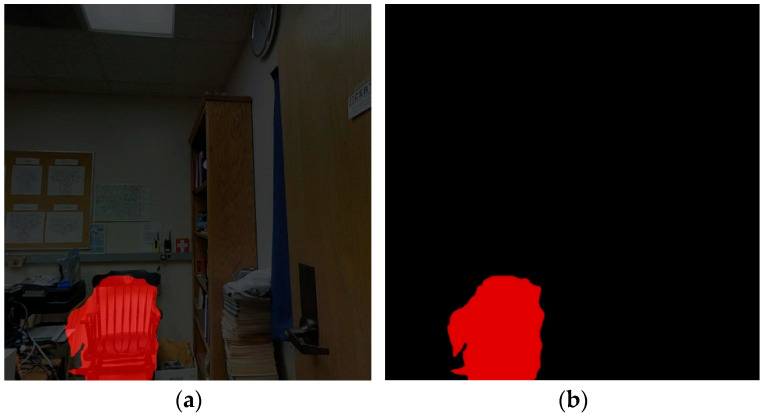
Prediction results show: (**a**) mixed mode (raw map + predicted map); (**b**) non-mixed mode.

**Figure 8:**
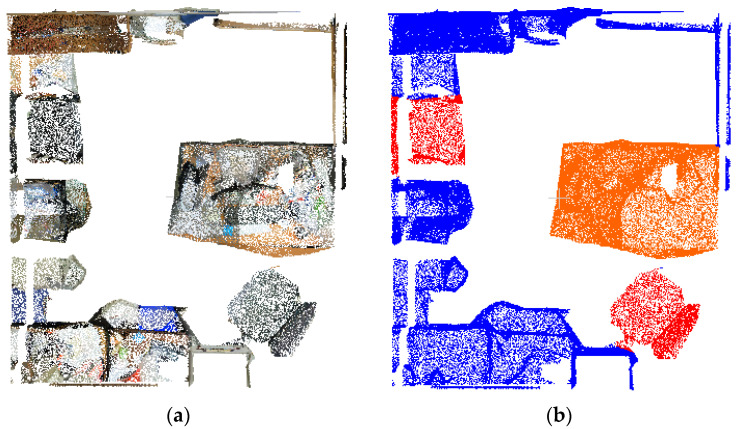
Top view of non-spatial layout point cloud data1 dataset (179,420 points): (**a**) color point cloud; (**b**) preliminary labeling of point clouds.

**Figure 9:**
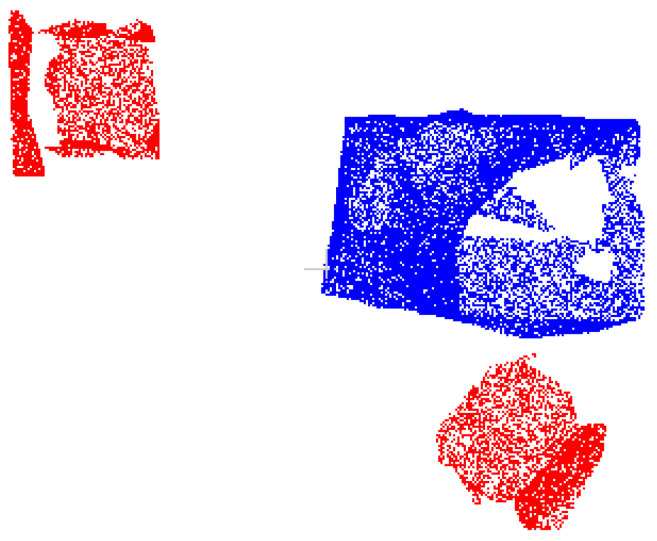
True value data2 dataset (36,400 points).

While the 2D-3D-S dataset provides 2D semantic labels, the self-collected real-world image data lack annotations. To avoid the time cost and expenses associated with manual labeling, this study directly employs the semantic segmentation model from Section 2.2 to identify objects in 2D-3D-S images (prediction results shown in Figure 7). For the label processing of predicted images, VOC-format color labels are mapped to the semantic category IDs of the office_7 dataset. The procedure entails the following steps: (1) projecting 2D semantic labels onto 3D point clouds, (2) extracting the RGB label values of target objects in the point cloud, and (3) assigning corresponding semantic category IDs. This yields the preliminarily labeled point cloud dataset data1 (Figure 8). For validation, ground-truth labels of tables and chairs are extracted from the office_7 dataset to construct the reference dataset data2 (Figure 9).

### 3.2. Adaptive DBSCAN Clustering with KD-Tree Acceleration

This section evaluates the impact of two adaptive parameter-setting strategies on improving point cloud label correction for semantic segmentation. The evaluation criteria for both strategies are outlined below:

Method 1: Global Parameter Optimization involves estimating a unified set of DBSCAN parameters (eps and MinPts) adaptively for the entire dataset (data1). This is achieved by processing the data label by label, with clustering operations facilitated by KD-tree acceleration for efficiency. Within each subset of labeled point clouds, the data are partitioned into multiple clusters. Clusters larger than a predefined size threshold are regarded as foreground objects, whereas smaller clusters are relabeled as background (label = 0, ClusterID = 0).

**Figure 10:**
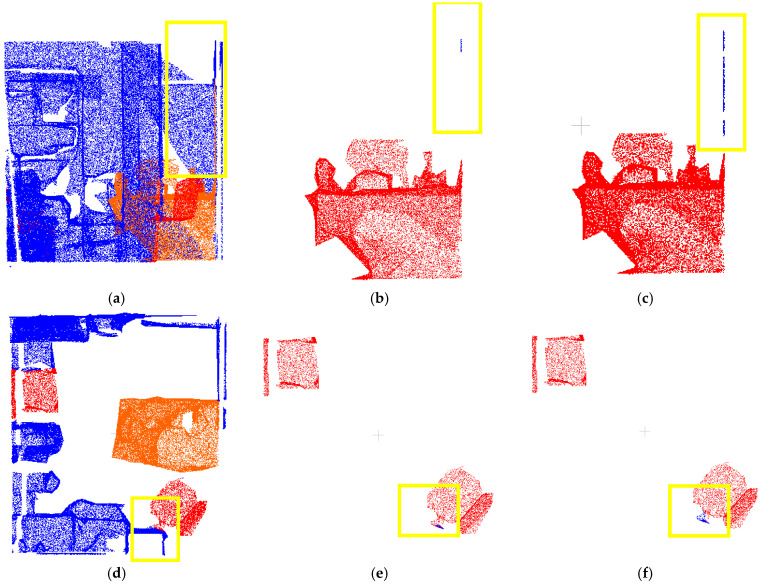
Comparison of calibration results of target point cloud with different parameter setting methods for data1 dataset: (**a**) data1 front view; (**b**) point cloud after correction (eps = 0.0534556, MinPts = 53); (**c**) point cloud after correction (eps = 0.0413169, MinPts = 36); (**d**) data1 top view; (**e**) point cloud after correction (eps = 0.0534556, MinPts = 53); (**f**) point cloud after correction (eps = 0.0379938, MinPts = 15).

Method 2: Category-Specific Parameter Optimization takes the following approach: the preliminarily labeled dataset (data1) is first divided into point clouds for each semantic category. Subsequently, adaptive DBSCAN parameters are calculated individually for each category. The clustering, accelerated again by KD-tree, is performed category-wise, with large clusters considered valid objects and small clusters discarded as the background (label = 0, ClusterID = 0). These processes are visually represented in Figure 10c,f.

#### 3.2.1. Analysis of Correction Effectiveness and Efficiency

**Figure 11:**
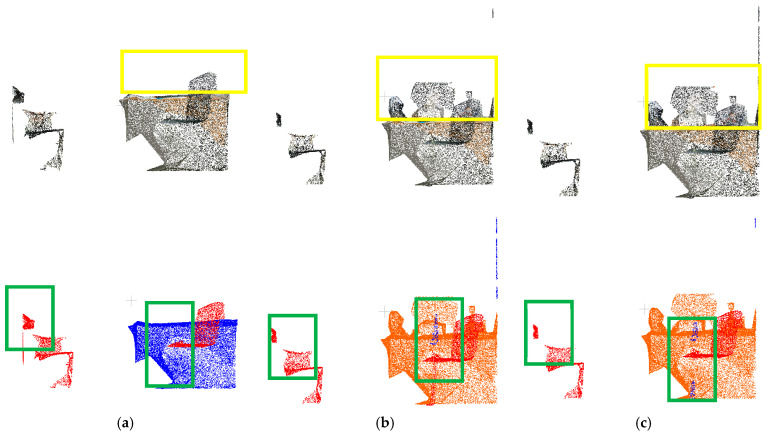
Comparison of data2 true value dataset and clustering optimization corrected results (green box part): (**a**) data2 front view; (**b**) results after calibration according to method 1; (**c**) results after calibration according to method 2.

Figure 10 demonstrates successful boundary-point label correction by both methods within the yellow annotated regions. Comparative analysis reveals significant differences in noise filtering due to parameter selection: while (b) and (c) show distinct noise-handling behaviors, (c) correctly identifies more boundary points as background rather than noise. Similarly, (e) and (f) confirm that parameter choices directly affect correction outcomes, with (f) properly classifying more boundary points as background. However, as shown in Figure 11, the initial annotations in dataset data1 only coarsely labeled two target categories, with table labels erroneously including cluttered objects (yellow box). The physical connections between targets and clutter led to suboptimal initial segmentation. Experimental results indicate that both methods exhibit substantial deviations from ground truth in boundary-point correction due to these initial labeling limitations. The parameter settings and time consumption data presented in Table 1 demonstrate that the total processing time of method 2 is significantly lower than that of method 1. Method 2 employs class-specific parameter optimization, where the parameter estimation is performed separately within each target point cloud category. This approach effectively reduces the neighborhood search range and computational load of distance measurements, thereby lowering the overall time complexity.

#### 3.2.2. Quantitative Analysis Results

Quantitative metrics in Table 2 reveal marginal differences in correction accuracy between the two parameter estimation methods. However, method 2 (class-specific parameter optimization) achieves comparable precision (↑73.83%) while reducing total computational time by 71% (12.1 s vs. 42.9 s). Leveraging this efficiency advantage, this study adopts method 2’s corrected labels and merges them with background points (label = 0) from dataset data1 to construct a refined dataset data3 for subsequent optimization experiments.

### 3.3. Point Cloud Regrowth for Semantic Segmentation Optimization

**Figure 12:**
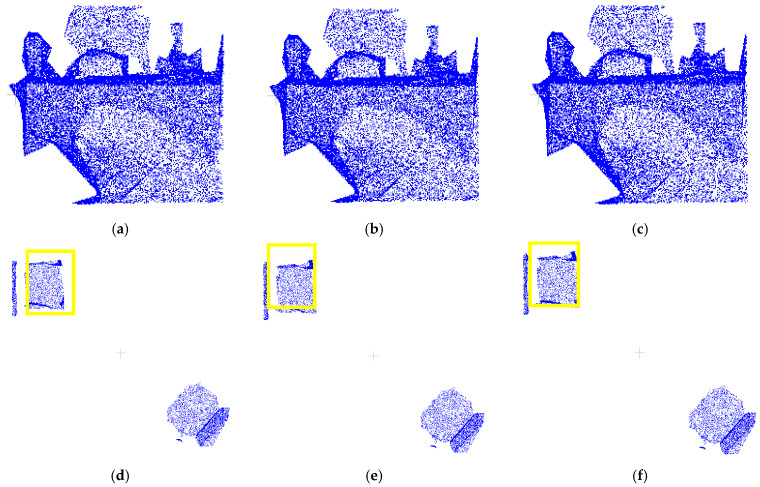
Comparison of the target point cloud regrowth correction results with different threshold settings $r_{1}$ for the data3 dataset: (**a**) target point cloud in data3; (**b**) target point cloud after regrowth; (**c**) target point cloud after regrowth; (**d**) target point cloud in data3; (**e**) target point cloud after regrowth; (**f**) target point cloud after regrowth.

After obtaining the cluster-optimized dataset data3, while mislabeled boundaries of target point clouds are improved, unannotated boundary points persist (see Figure 12a,d). To address this, we designed a comparative experiment based on the algorithm in Section 2.4.2: (1) using a unified fixed radius threshold (0.05 m) and (2) employing the adaptively estimated optimal eps value as the radius threshold.

Visual comparisons in Figure 12 show negligible differences between the fixed-threshold (b, e) and adaptive (c, f) schemes. For objective evaluation, Table 3 presents quantitative metrics comparing both approaches. The analysis reveals minor performance gaps between fixed and adaptive thresholds, with both achieving acceptable correction accuracy. However, given the inherent density variations across different object categories in practical applications, this study adopts the adaptive thresholding algorithm from Section 2.4.2 for unannotated point recall. This approach automatically optimizes parameters based on each category’s spatial distribution, minimizing manual tuning while enhancing robustness and generalizability.

**Figure 13:**
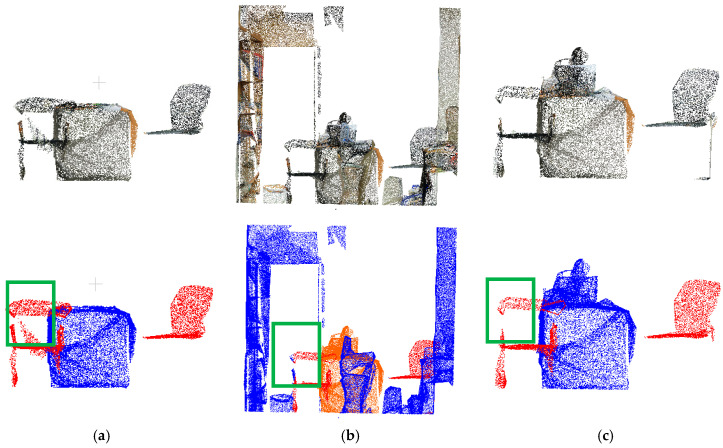
Comparison of the data2 true value dataset, the data3 clustering optimized dataset, and the regrowth optimized corrected results (green box part): (**a**) data2 left side view; (**b**) data3 left side view; (**c**) regrowth optimization results.

Figure 13 compares the ground truth (data2), cluster-optimized dataset (data3), and regrowth optimization results in the green bounding box regions. The visual analysis demonstrates that the regrowth algorithm effectively improves upon the cluster-optimized results by successfully recovering previously unannotated points of target objects while enhancing boundary completeness. As evidenced by the metrics in Table 4, the recall rate remains consistent across the first two processing phases, indicating that the algorithm maintains the number of correctly predicted samples while eliminating mislabeled points. In the third phase, all metrics except the F1-score show marginal improvements. This enhancement stems from the regrowth optimization successfully recovering partial point clouds that intrinsically belong to target objects, thereby completing missing labels and increasing the count of accurately predicted samples. The results demonstrate that the regrowth optimization effectively improves annotation completeness in point cloud data.

### 3.4. Real-World Validation

**Figure 14:**
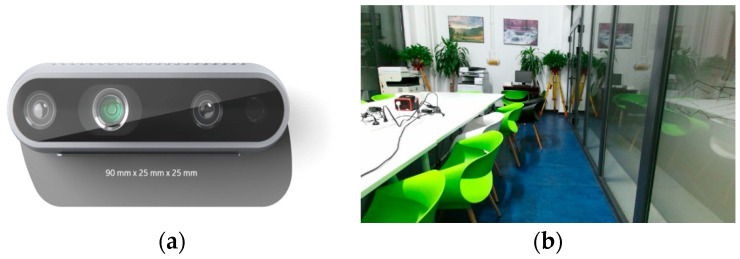
Experimental equipment and scene: (**a**) the depth camera used in this research; (**b**) the experimental scenario of this research.

To validate the feasibility of the image-assisted semantic annotation method for indoor SLAM point clouds proposed in previous sections, experimental data were collected from a real indoor environment using a calibrated RealSense D435 depth camera (Intel, Guangdong, China) and preprocessed accordingly. The experiment was conducted in an office at the School of Geomatics, Beijing University of Civil Engineering and Architecture [29], as shown in Figure 14. During RGB-D SLAM operation with the depth camera, the target room was selected as the core operational area to meet the requirements for backend optimization and loop closure detection. The robotic platform performed “return-to-origin” movements to correct the overall trajectory shape and reduce accumulated drift errors.

The image semantic segmentation model was then applied to generate predictions, and labels were transferred to the point cloud data based on the image-to-point cloud mapping relationship to obtain preliminary annotation results. To further optimize the annotations and improve segmentation accuracy, cluster-based processing and regrowth post-processing were performed on target objects to address mislabeled and unlabeled cases, followed by metric evaluation. All experiments were conducted on a computer equipped with an Intel i7-4790 CPU (Intel, Santa Clara, CA, USA) and 16 GB RAM.

#### 3.4.1. Point Cloud Preprocessing and Preliminary Semantic Segmentation Labeling

**Figure 15:**
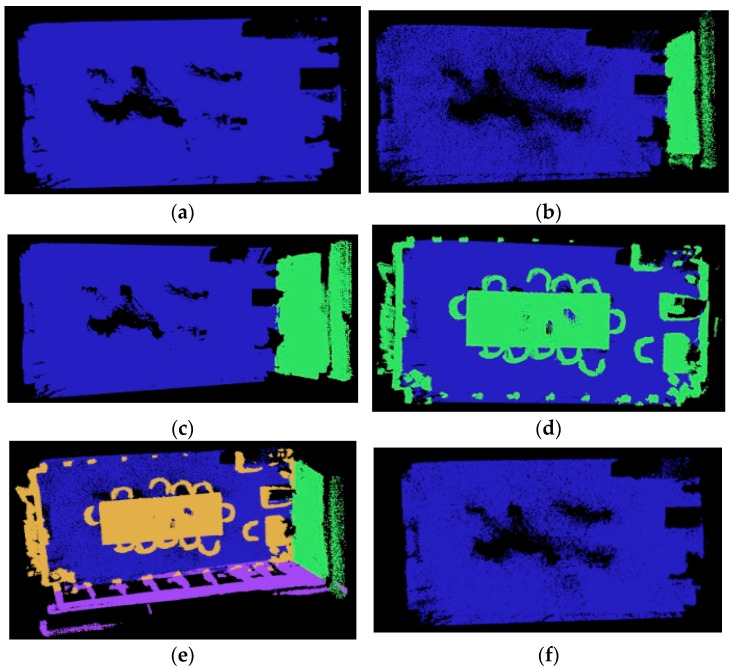
Large area planar point cloud segmentation comparison experiment: (**a**) experiment 1 (D = 0.04); (**b**) experiment 2 (D = 0.08); (**c**) experiment 3 (D = 0.10); (**d**) experiment 4 (D = 0.12); (**e**) experiment 5/6 (Min = 250,000); (**f**) experiment 7 (Min = 600,000).

In the analysis of the preprocessed point cloud dataset, the wall data collection is incomplete. Consequently, when employing the RANSAC-based multiplanar segmentation algorithm for filtering out large-scale spatial layout point clouds, careful consideration must be given to the choice of the threshold value. For this purpose, we chose the threshold value based on the grid search method, as depicted in Table 5. Min in Table 5 indicates the minimum internal point threshold, and Max indicates the maximum threshold for the number of remaining exterior. Subsequently, a comparison experiment was conducted, and the results are shown in Figure 15.

**Figure 16:**
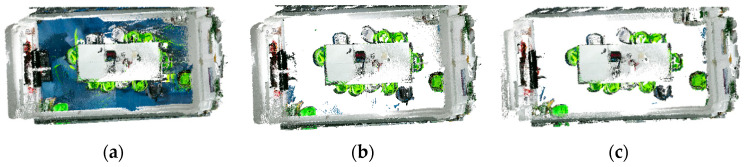
Point cloud preprocessing: (**a**) manually processed point cloud; (**b**) point cloud after separating ground points; (**c**) point cloud filtered by the SOR algorithm.

**Figure 17:**
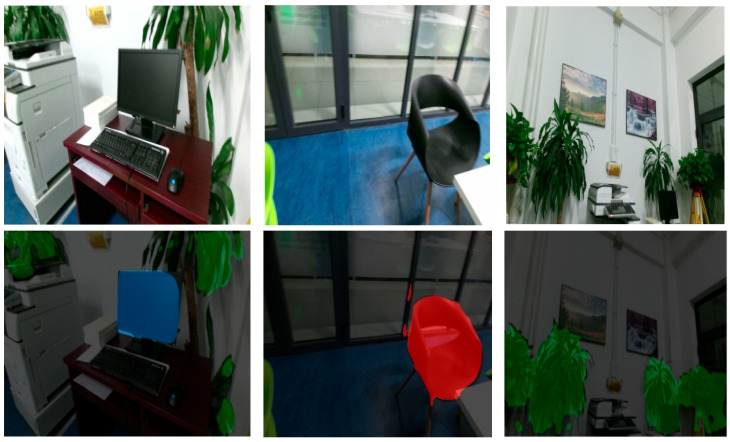
Image semantic segmentation results.

**Figure 18:**
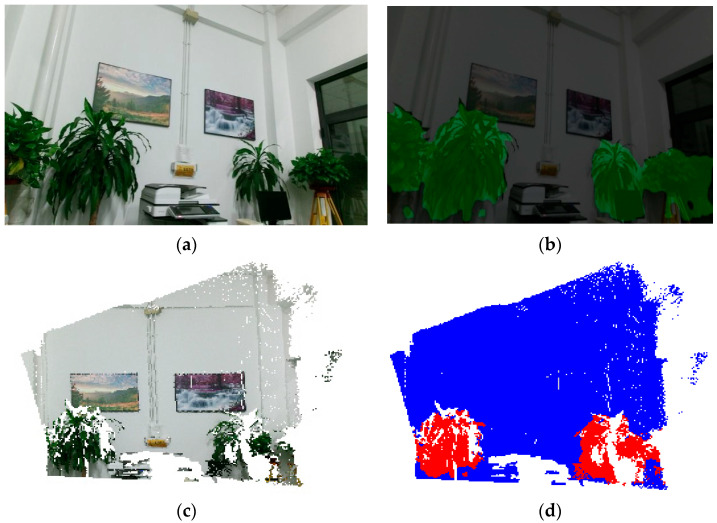
Comparison of semantic segmentation results on keyframes with the DeepLabV3+ model. This keyframe image displays the recognition results of green plants in an office setting, and the image was converted to a point cloud through back-projection: (**a**) single keyframe image; (**b**) semantic segmentation of images before improvement; (**c**) color point cloud generated from a single keyframe image; (**d**) semantic labeling of point clouds (with background).

Figure 16 demonstrates the application of a Statistical Outlier Removal (SOR) filter [30,31] to effectively eliminate anomalous data points in the point cloud. Table 6 presents the semantic label categories for indoor point cloud maps, which were designed by integrating target classes from the PASCAL VOC image dataset [32] and the S3DIS indoor point cloud dataset. Using the DeepLabV3+ semantic segmentation model, predictions were generated for 420 keyframe images captured in this study, yielding 2D semantic segmentation maps (exemplified in Figure 17 for displays, chairs, and plants). Keyframe images are processed on the CPU only for about 2.32 s each. The process of labeling the data followed this sequence: Calibrated SLAM systems generated synchronized image-point cloud pairs, which included pose information. This information allowed for the accurate projection of 2D semantic labels onto the corresponding 3D point clouds. Figure 18 displays the preliminary semantic annotation results for a single-frame target point cloud.

**Figure 19:**
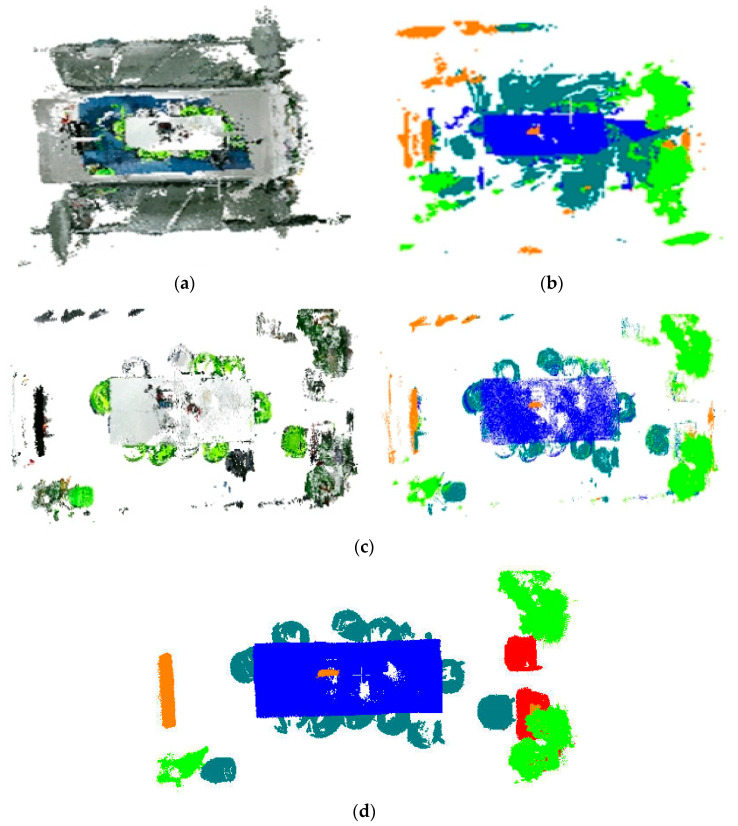
Preliminary annotation results of target point cloud (209,294 points) and true value label: (**a**) target color point cloud; (**b**) target semantic point cloud; (**c**) target point cloud after processing; (**d**) true value label.

Figure 19 demonstrates the effective separation of target objects in the processed point cloud. This point cloud shows distinct objects such as the blue table, dark green chair, cyan potted plant, and orange-red monitor. Additionally, it reveals a marked decrease in mislabeled ground points and an overall simplification of the data. To assess the quantitative performance of the semantic annotation at each phase of the processing, we constructed a comprehensive ground truth dataset that annotates four specific categories of objects: tables, chairs, plants, and monitors. These annotations are based on preprocessed point clouds, as depicted in Figure 19d. Quantitative analysis of the preliminary annotation results (Table 7) reveals that among the four categories, only the potted plant achieves satisfactory performance metrics, while the remaining categories exhibit suboptimal annotation accuracy.

#### 3.4.2. Point Cloud Clustering of Target Objects

**Figure 20:**
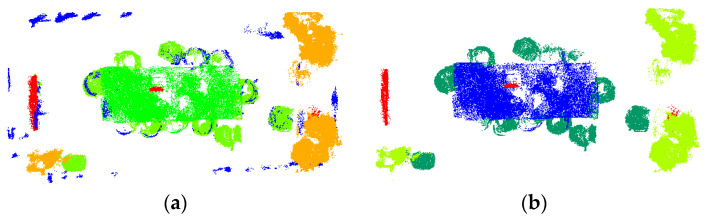
Target point cloud clustering optimization annotation results (192,988 points in total): (**a**) after correction (blue is the background points that were removed); (**b**) target point cloud after clustering correction.

The results in Figure 20 demonstrate effective elimination of discrete non-target clusters. Table 8 details the parameter configurations and corresponding processing times employed in the experiments. The quantitative metrics in Table 9 reveal that the optimized processing yields marginal improvements in accuracy and precision, successfully correcting a portion of mislabeled samples while maintaining stable recall rates.

#### 3.4.3. Target Point Cloud Regrowth

**Figure 21:**
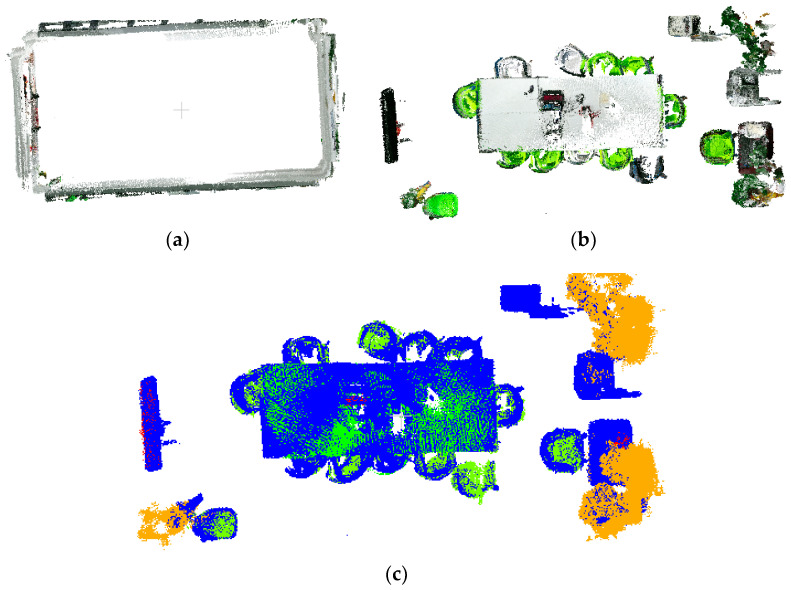
Point cloud after separating the wall and unmarked point cloud: (**a**) wall point cloud; (**b**) unlabeled point cloud after manual processing; (**c**) introducing the unmarked point cloud (956,426 points in total).

**Figure 22:**
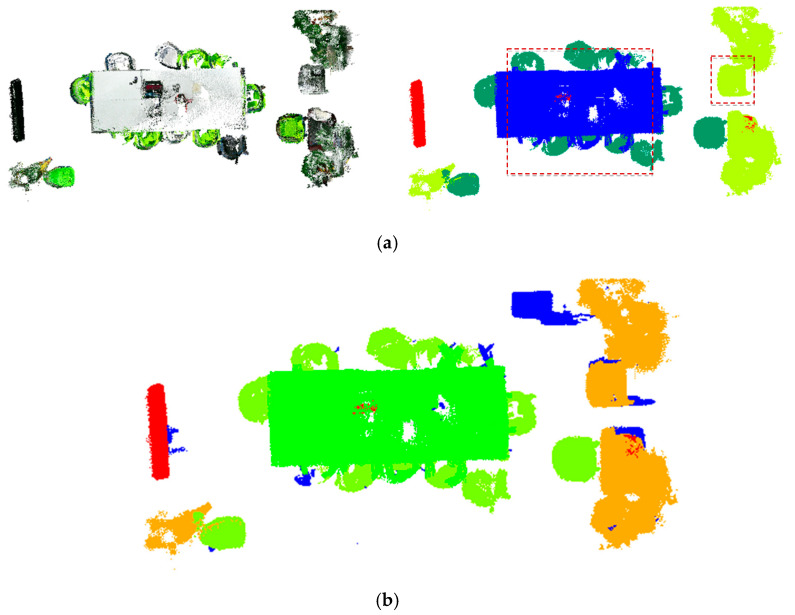
Regeneration optimization annotation results and corrected point cloud: (**a**) target point cloud regeneration optimization annotation results (793,503 points in total); (**b**) corrected point cloud (with blue background points).

The regrown point cloud dataset, as presented in Figure 21c, is a result of merging the optimized target point cloud from Figure 20 with the unannotated point cloud from Figure 21b. This dataset contains a total of 956,426 data points, where the newly incorporated points are visually distinguished in blue. The merging process, as depicted in Figure 22a, significantly increases the scale of the target object point clouds. However, it is important to note that this expansion also brings about an increased probability of mislabeling.

Table 10 details the key parameter configurations and point cloud statistics during the regrowth process. Cross-analysis of Figure 22b and Table 10 reveals that the regrown table point cloud erroneously incorporates background points from adjacent chairs, while the monitor objects demonstrate near-ideal calibration results.

Quantitative evaluation metrics in Table 11 indicate substantial improvements across all categories compared to the initial annotations. When compared to the mislabel-corrected results, all categories except potted plants show notable enhancements. The plant category’s stagnation is attributed to its complex geometric and textural characteristics, which challenge the current regrowth algorithm’s capability.

#### 3.4.4. Discussion

**Figure 23:**
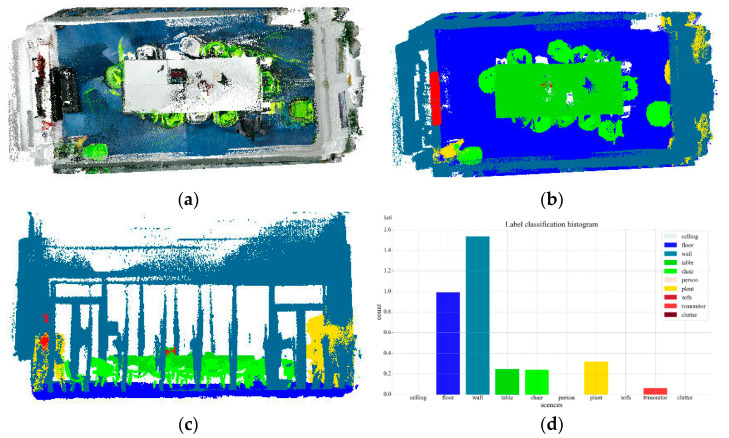
Semantic annotation of indoor 3D scenes: (**a**) color information; (**b**) semantic information top view; (**c**) semantic information front view; (**d**) label classification histogram.

Table 12 compares the evaluation metrics across all correction phases for each category. Experimental analysis demonstrates that Phase II achieves significantly improved accuracy over Phase I, confirming the effective correction of mislabeled instances, though with a slight decrease in recall rate. The stable F1-score further verifies the overall reliability of the mislabel correction approach. Phase III shows substantial improvements across all metrics compared to Phase II, demonstrating clear optimization effects. Finally, by integrating floor and wall point clouds, the complete semantic annotation of the indoor scene is accomplished, as shown in Figure 23.

## 4. Discussion and Conclusions

This study proposes a 3D point cloud semantic annotation framework integrating the DeepLabV3+ semantic segmentation network with automatic label correction algorithms, aiming to address the accuracy and efficiency challenges in semantic annotation for visual SLAM systems in indoor environments. By combining deep learning with point cloud optimization algorithms, the framework achieves automated, high-precision semantic annotation from 2D images to 3D point clouds. The research employs a lightweight MobileNetV3 as the backbone network for DeepLabV3+, maintaining segmentation accuracy while reducing computational overhead. Furthermore, an adaptive DBSCAN clustering correction algorithm and a Euclidean distance-driven point cloud regrowth optimization method are proposed, significantly enhancing annotation completeness and accuracy.

For semantic segmentation, DeepLabV3+ achieved an initial annotation accuracy of 72.8% on the 2D-3D-S dataset. To enhance annotation quality, an adaptive DBSCAN clustering algorithm with K-Average Nearest Neighbor (KANN)-based automatic parameter estimation and KD-tree acceleration was employed. In this methodology, the accuracy rate of correcting mislabeled data was notably enhanced to 73.83%, concurrently achieving a reduction of 71% in the overall processing duration. This advancement substantially boosted clustering efficiency and significantly curtailed the need for manual intervention. To address missing annotations, a dynamic radius threshold-based point cloud regrowth optimization strategy was designed, which significantly increased the final F1-score from 0.38 to 0.98 and achieved a recall rate of 99.8%. Experimental results demonstrate that the proposed method effectively separates target objects such as tables, chairs, and monitors, generating high-quality semantic point cloud maps. This provides a reliable environmental understanding solution for indoor robot navigation.

However, this study still presents several limitations: (1) The initial annotation quality is constrained by 2D segmentation performance, leading to significant errors in complex scenes; (2) while our RANSAC-based plane detection relies on manual parameter tuning, recent heuristics like BAS-ADAM [33] could optimize this process; (3) the point cloud regrowth phase exhibits suboptimal efficiency when processing large-scale datasets; and (4) the current method is designed solely for RGB-D data, lacking multi-sensor fusion capabilities. Future research will prioritize addressing these challenges to enhance the method’s practicality and generalization, ultimately promoting its applications in autonomous driving, AR/VR, and related fields.

## Figures and Tables

**Table 1:** Parameter and runtime comparison of two clustering strategies: (1) global optimization (method 1) and (2) category-specific optimization (method 2).

	Eps Value	MinPts Value	Param Time (s)	Table Time (s)	Chair Time (s)
Method 1	0.0534556	53	42.587	0.21	0.066s
Method 2	0.0413169	36	8.731	0.152	\
	0.0379938	15	3.192	\	0.033

**Table 2:** Evaluation of calibration result indicators for different target point clouds.

Model	Category	Eps	MinPts	Accuracy (%)	Precision (%)	Recall (%)	F1 Value
DeepLabV3+	Table	\	\	68.73	70.95	95.65	0.81
	Chairs	\	\	86.24	98.07	87.73	0.93
	All	\	\	72.80	76.79	93.33	0.84
DeepLabV3+-Adaptive DBSCAN Method 1	Table	0.0534556	53	69.66	71.94	95.65	0.82
	Chairs	0.0534556	53	86.46	98.34	87.73	0.93
	All	\	\	73.59	77.68	93.33	0.85
DeepLabV3+-Adaptive DBSCAN Method 2	Table	0.0413169	36	69.67	71.95	95.65	0.82
	Chairs	0.0379938	15	87.58	99.81	87.73	0.93
	All	\	\	**73.83**	**77.94**	93.33	0.85

**Table 3:** Evaluation of calibration results metrics for target point clouds with different threshold settings.

Category	Radius Threshold	Accuracy (%)	Precision (%)	Recall (%)	F1 Value
Table	*r*_1_ = 0.05	69.67	71.95	95.65	0.82
	*r*_1_ = 0.0413169	69.67	71.95	95.65	0.82
Chairs	*r*_1_ = 0.05	87.86	99.81	88.01	0.93
	*r*_1_ = 0.0379938	87.86	99.81	88.01	0.93

**Table 4:** Evaluation of calibration result indicators for all categories of point clouds at different phases.

Model	Accuracy (%)	Precision (%)	Recall (%)	F1 Value
DeepLabV3+ (Phase I)	72.80	76.79	93.33	0.84
DeepLabV3+-Adaptive DBSCAN Method 2 (Phase II)	73.83	77.94	93.33	0.85
DeepLabV3+-Adaptive DBSCAN Method 2-Regrowth Optimization (Phase III)	**73.89**	**77.95**	**93.41**	**0.85**

**Table 5:** Comparison of planar segmentation thresholds (300 iterations).

Number	D (m)	Min	Max	Points	Planes
1	**0.04**	500,000	100,000	750,784	1
2	**0.08**	500,000	100,000	1,496,337	2
3	**0.10**	500,000	100,000	1,584,185	2
4	**0.12**	500,000	100,000	1,592,514	2
5	0.10	**250,000**	100,000	2,327,828	4
6	0.10	250,000	**1,500,000**	2,327,828	4
7	**0.10**	**600,000**	**1,500,000**	**993,814**	**1**

**Table 6:** Label.

Category	Ceiling	Floor	Wall	Table	Chair	Person	Plant	Sofa	Tv Monitor	Clutter/Bottle
Tag	1	2	3	4	5	6	7	8	9	10

**Table 7:** Evaluation of initial labeled target point cloud metrics.

Category	Accuracy (%)	Precision (%)	Recall (%)	F1 Value
Table	16.54	89.10	16.88	0.28
Chairs	15.17	70.21	16.22	0.26
Plants	63.32	93.62	66.17	0.78
Monitors	4.89	46.05	5.19	0.09
All	23.55	89.29	24.23	0.38

**Table 8:** Parameter estimation and time-consuming statistics for point cloud clustering of target objects.

Category	Points	Eps Value	MinPts Value	Parameter Estimation Time (s)	Clustering Time (s)	Total Correction Time (s)	Percentage of Corrected Points (%)
Table	34,107	0.125936	23	14.126	0.667	14.793	94.44
Chairs	45,290	0.141228	50	40.112	1.229	41.341	92.04
Plants	117,827	0.20429	18	1130.32	19.581	1149.901	97.84
Display	10,002	0.115843	36	2.036	0.08	2.116	51.84
NULL	2068	\	\	\	\	\	\
Total	209,294	\	\	1186.594	21.557	1208.151	92.21

**Table 9:** Evaluation of target point cloud metrics after mislabeling correction.

Model	Category	Accuracy (%)	Precision (%)	Recall (%)	F1 Value
DeepLabV3+	Table	16.54	89.10	16.88	0.28
	Chairs	15.17	70.21	16.22	0.26
	Plants	63.32	93.62	66.17	0.78
	Monitors	4.89	46.05	5.19	0.09
	All	23.55	89.29	24.23	0.38
DeepLabV3+-Adaptive DBSCAN	Table	16.73	94.92	16.88	0.29
	Chairs	15.42	77.05	16.17	0.27
	Plants	64.29	95.77	66.17	0.78
	Monitors	4.95	52.08	5.19	0.09
	All	23.36	92.30	23.82	0.38

**Table 10:** Parameter setting and point count for point cloud regrowth of target objects.

Category	Points After Clustering	Object Number	r1 Value	r2 Value	Points After Regrowth
Table	32,209	1	0.125936	2.16807	221,940
Chairs	14,161	1	0.141228	1.69852	177,335
	9008	2		1.08328	
	5127	3		0.562582	
	5075	4		0.772056	
	4688	5		1.0368	
	3625	6		0.578157	
Plants	52,450	1	0.20429	1.65155	317,883
	52,026	2		1.58817	
	10,809	3		1.14409	
Monitors	3359	1	0.115843	0.850999	76,323
	1382	2		0.450876	
	444	3		0.242609	

**Table 11:** Evaluation of unlabeled corrected target point cloud metrics.

Model	Category	Accuracy (%)	Precision (%)	Recall (%)	F1 Value
DeepLabV3+	Table	16.54	89.10	16.88	0.28
	Chairs	15.17	70.21	16.22	0.26
	Potted Plants	63.32	93.62	66.17	0.78
	Monitors	4.89	46.05	5.19	0.09
	All	23.55	89.29	24.23	0.38
DeepLabV3+-Adaptive DBSCAN	Table	16.73	94.92	16.88	0.29
	Chairs	15.42	77.05	16.17	0.27
	Plants	64.29	95.77	66.17	0.78
	Monitors	4.95	52.08	5.19	0.09
	All	**23.36**	**92.30**	**23.82**	0.38
DeepLabV3+-Adaptive DBSCAN-Regrowth Optimization	Table	80.56	81.03	99.29	0.89
	Chairs	75.10	91.53	80.71	0.86
	Plants	51.53	51.86	98.79	0.68
	Monitors	80.40	99.34	80.84	0.89
	All	**96.82**	**97.00**	**99.80**	**0.98**

**Table 12:** Evaluation of calibration results metrics for all categories of point clouds at different phases.

Model	Accuracy (%)	Precision (%)	Recall (%)	F1 Value
DeepLabV3+ (Phase I)	23.55	89.29	24.23	0.38
DeepLabV3+-Adaptive DBSCAN (Phase II)	23.36	92.30	23.82	0.38
DeepLabV3+-Adaptive DBSCAN-Regrowth Optimization (Phase III)	96.82	97.00	99.80	0.98

## Data Availability

The data are contained within the article.
